# Efficacy of Kisspeptin-54 to Trigger Oocyte Maturation in Women at High Risk of Ovarian Hyperstimulation Syndrome (OHSS) During In Vitro Fertilization (IVF) Therapy

**DOI:** 10.1210/jc.2015-2332

**Published:** 2015-07-20

**Authors:** Ali Abbara, Channa N. Jayasena, Georgios Christopoulos, Shakunthala Narayanaswamy, Chioma Izzi-Engbeaya, Gurjinder M. K. Nijher, Alexander N. Comninos, Deborah Peters, Adam Buckley, Risheka Ratnasabapathy, Julia K. Prague, Rehan Salim, Stuart A. Lavery, Stephen R. Bloom, Matyas Szigeti, Deborah A. Ashby, Geoffrey H. Trew, Waljit S. Dhillo

**Affiliations:** Department of Investigative Medicine (A.A., C.N.J., S.N., C.I.-E., G.M.K.N., A.N.C., D.P., A.B., R.R., J.K.P., S.R.B., W.S.D.), Imperial College London, Hammersmith Hospital, London, W12 0NN, United Kingdom; IVF Unit (G.C., R.S., S.A.L., G.H.T.), Hammersmith Hospital, London, W12 0HS, United Kingdom; and Imperial Clinical Trials Unit (M.S., D.A.A.), Imperial College London, St Mary's Hospital, Norfolk Pl, London, W2 1PG, United Kingdom

## Abstract

**Context:**

In vitro fertilization (IVF) treatment is an effective therapy for infertility, but can result in the potentially life-threatening complication, ovarian hyperstimulation syndrome (OHSS).

**Objective:**

This study aimed to investigate whether kisspeptin-54 can be used to effectively and safely trigger oocyte maturation in women undergoing IVF treatment at high risk of developing OHSS.

**Setting and Design:**

This was a phase 2, multi-dose, open-label, randomized clinical trial of 60 women at high risk of developing OHSS carried out during 2013–2014 at Hammersmith Hospital IVF unit, London, United Kingdom.

**Intervention:**

Following a standard recombinant FSH/GnRH antagonist protocol, patients were randomly assigned to receive a single injection of kisspeptin-54 to trigger oocyte maturation using an adaptive design for dose allocation (3.2 nmol/kg, n = 5; 6.4 nmol/kg, n = 20; 9.6 nmol/kg, n = 15; 12.8 nmol/kg, n = 20). Oocytes were retrieved 36 h after kisspeptin-54 administration, assessed for maturation, and fertilized by intracytoplasmic sperm injection with subsequent transfer of one or two embryos. Women were routinely screened for the development of OHSS.

**Main Outcome Measure:**

Oocyte maturation was measured by oocyte yield (percentage of mature oocytes retrieved from follicles ≥ 14 mm on ultrasound). Secondary outcomes include rates of OHSS and pregnancy.

**Results:**

Oocyte maturation occurred in 95% of women. Highest oocyte yield (121%) was observed following 12.8 nmol/kg kisspeptin-54, which was +69% (confidence interval, −16–153%) greater than following 3.2 nmol/kg. At all doses of kisspeptin-54, biochemical pregnancy, clinical pregnancy, and live birth rates per transfer (n = 51) were 63, 53, and 45%, respectively. Highest pregnancy rates were observed following 9.6 nmol/kg kisspeptin-54 (85, 77, and 62%, respectively). No woman developed moderate, severe, or critical OHSS.

**Conclusion:**

Kisspeptin-54 is a promising approach to effectively and safely trigger oocyte maturation in women undergoing IVF treatment at high risk of developing OHSS.

One in seven couples in the United Kingdom are affected by infertility (1). The inability to conceive can be devastating and has important implications for mental, social, and reproductive health. The use of assisted reproductive techniques to help such couples is increasing world wide, with more than 2% of babies born in the United Kingdom in 2012 being conceived through in vitro fertilization (IVF) treatment (1). However, IVF treatment can result in the potentially life-threatening condition, ovarian hyperstimulation syndrome (OHSS). This is one of the most common and perilous complications of IVF treatment (2) and can result in massive ovarian enlargement, ascites, hydrothorax, renal failure, acute respiratory distress syndrome, and even death (3). Although the risk of severe OHSS is approximately 2% in an unselected population (4), in patient groups with risk factors for OHSS such as polycystic ovarian syndrome, the risk of OHSS is increased 5-fold (5) and severe OHSS has been reported to occur in as much as a quarter of patients undergoing IVF treatment (6).

The major cause of OHSS is the pharmacological use of human chorionic gonadotropin (hCG) to induce oocyte maturation in current IVF protocols (4). The physiological stimulus for oocyte maturation in a normal menstrual cycle is the LH surge that has a duration of approximately 48 hours (7). hCG has LH-like activity that persists in the circulation for up to a week following administration (8) and thus can result in excessive ovarian stimulation and the occurrence of OHSS. A number of strategies such as the use of GnRH antagonist cycles with GnRH agonist triggering, segmentation, in vitro maturation, dopamine agonists, iv albumin, metformin, coasting, and cycle cancellation have been employed in an attempt to reduce the risk of OHSS; however, a single approach has not garnered universal agreement in clinical practice (9, 10).

Thus, it is important to explore novel strategies to avoid OHSS in otherwise healthy women seeking fertility treatment while maintaining good implantation rates. A more physiological stimulus for oocyte maturation would avoid this dangerous complication and improve the safety of IVF treatment (11). Kisspeptin is a hormone that offers a novel approach to trigger oocyte maturation during IVF treatment (12). We recently reported that kisspeptin-54 can induce high rates of oocyte maturation in women with normal ovarian reserve undergoing IVF treatment (12). However, it is not known whether kisspeptin can safely trigger oocyte maturation in women at high risk of OHSS. Kisspeptin stimulates the release of endogenous GnRH from the hypothalamus and the consequent release of LH and FSH (13). Hence, kisspeptin induces an LH surge that is dependent upon the patient's individual endogenous GnRH/gonadotropin reserves and thus should prevent excessive stimulation of the ovaries. Based on this unique mechanism of action, we hypothesized that kisspeptin-54 would effectively trigger oocyte maturation, yet also have a low risk of inducing OHSS.

To test this hypothesis, we conducted a phase 2 randomized clinical trial to investigate the efficacy and safety of kisspeptin-54 to trigger oocyte maturation in women undergoing IVF treatment at high risk of developing OHSS.

## Materials and Methods

### Study approval

The Hammersmith and Queen Charlotte's Research Ethics Committee, London, United Kingdom, approved this study (Reference: 10/H0707/2). The study was carried out in the IVF Unit at Hammersmith Hospital under a license from the United Kingdom Human Fertilization and Embryology Authority. Written informed consent was obtained from all subjects prior to inclusion in the study. Approval of this study as a Clinical Trial of an Investigative Medicinal Product was granted by the Medicines and Healthcare Products Regulatory Agency, UK. The study was registered on the National Institutes of Health Clinical Trials database (ClinicalTrials.gov NCT01667406). The study was performed in accordance with the Declaration of Helsinki.

### Subjects

Seventy-one women requiring IVF treatment for infertility at Hammersmith Hospital, London, United Kingdom were screened for participation between October 2013 and August 2014. Eligible patients received a single treatment cycle; all treatment costs for the cycle were covered by study participation. We aimed to recruit women at high risk of developing OHSS during IVF treatment. Previous work has shown that women with either a serum anti-Müllerian hormone (AMH) greater than 40 pmol/L, or total ovarian antral follicle count (AFC) greater than 23 on ultrasound have a high risk of developing OHSS during IVF treatment (14, 15). The inclusion criteria were serum AMH at least 40 pmol/L (> 5.6 ng/mL) or total AFC greater than 23, age 18–34 years, early follicular phase serum FSH less than or equal to 12 mIU/mL, both ovaries intact, and body mass index (BMI) 18–29 kg/m^2^ (2). Exclusion criteria were moderate/severe endometriosis or poor response to more than one previous cycle of IVF treatment.

### Study outcomes

The primary outcome was oocyte maturation. This was assessed by oocyte yield (percentage of mature [metaphase 2; M2] oocytes collected from the number of follicles ≥ 14 mm on final ultrasound scan prior to kisspeptin-54 trigger administration). Oocytes were independently classified as M2 by presence of the first polar body and round ooplasm by an embryologist blinded to the dose of kisspeptin-54 administered (11).

Secondary outcomes were the occurrence of OHSS, fertilization rate (percentage of M2 oocytes that fertilize to form two pronuclear [2PN] zygotes following intracytoplasmic injection with sperm [ICSI]), embryo formation and quality, biochemical pregnancy rate (serum βhCG > 10 mIU/mL 11 d after embryo transfer), and clinical pregnancy rate (intrauterine gestational sac with heartbeat on ultrasound at 6 weeks' gestation).

### Randomization and masking

**Figure 1.**
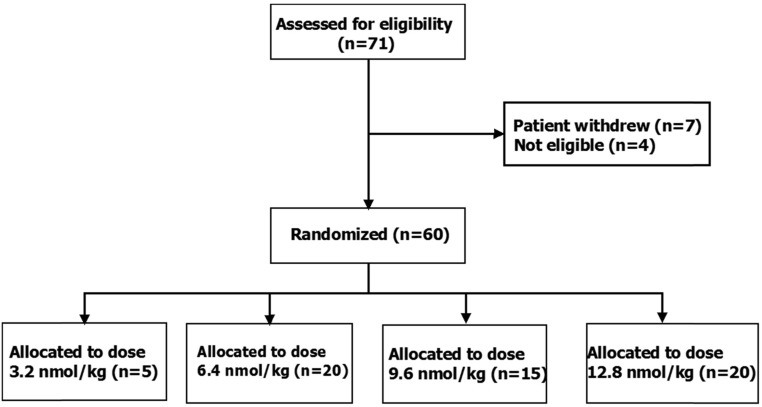
Patient flow diagram showing the number of patients assessed for eligibility, study enrolment, and kisspeptin-54 dose allocation. The study had a prospective adaptive design whereby the first 15 patients were randomly assigned 1:1:1 to receive kisspeptin-54 at doses of 3.2, 6.4, or 12.8 nmol/kg (n = 5 per group) to trigger oocyte maturation during IVF treatment. After interim analysis of oocyte maturation, subsequent patients were randomly assigned 1:1:1 to receive 6.4, 9.6, or 12.8 nmol/kg (n = 15 per group). No patients were lost to followup and no patients discontinued the intervention.

To identify the optimal dose of kisspeptin-54 in women at high risk of overstimulation while minimizing the number of women exposed to ineffective doses, an adaptive design for dose allocation was prospectively employed. Thus, the protocol stipulated an interim analysis by two clinical investigators independent of the study team to occur after the first 15 participants had been equally randomly assigned to receive one of three doses of kisspeptin-54 (3.2, 6.4, or 12.8 nmol/kg; n = 5 per group). If highest rates of oocyte maturation were encountered at 3.2 nmol/kg, then further patients would be randomly assigned to receive 1.6, 3.2, 6.4, or 9.6 nmol/kg (n = 15 per group). If highest rates of oocyte maturation were encountered at 6.4 or 12.8 nmol/kg, then further patients would be randomized to receive 6.4, 9.6, or 12.8 nmol/kg (n = 15 per group). The number of patients assessed for eligibility, study enrolment, and dose allocation are shown in Figure 1. Randomization was performed using printed lists that were prepared by the trial statistician using NQuery (http://www.statsols.com/products/nquery-advisor-nterim/) at the Imperial Clinical Trials Unit. Participants, embryologists, and the clinicians who carried out oocyte retrieval were all blinded to the dose allocation.

### Protocol

**Figure 2.**
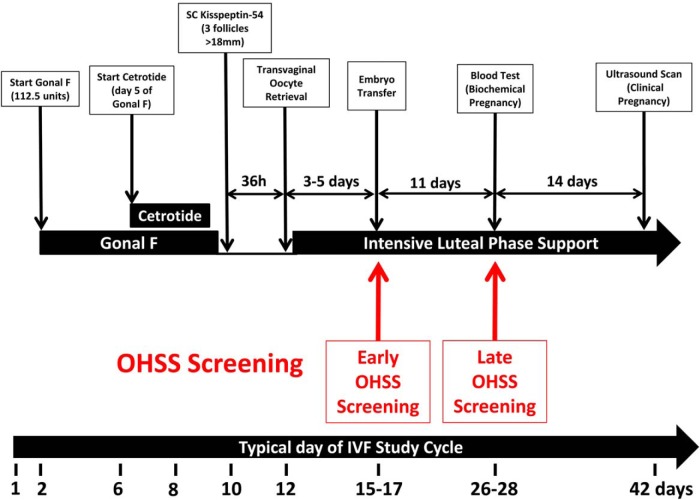
IVF study protocol using kisspeptin-54 to trigger oocyte maturation. The timeline shows the day of menstrual cycle for a typical patient. On day 2 or 3 of the menstrual cycle, daily sc recombinant FSH (Gonal F, 112.5 IU) was commenced. Daily GnRH antagonist injections (cetrotide, 0.25 mg) were commenced after 5 d of recombinant FSH injections. If serum LH was undetectable (< 0.5 IU/L) on day 7 of recombinant FSH injections, the dose of cetrotide was halved to 0.125 mg daily. When at least three ovarian follicles ≥18 mm diameters were visible on ultrasound, a sc bolus injection of kisspeptin-54 (3.2, 6.4, 9.6, or 12.8 nmol/kg) was administered to trigger oocyte maturation (between 2030 and 2300 h). Injections of GnRH antagonist and FSH were stopped 24 and 12 h prior to kisspeptin administration, respectively. Transvaginal ultrasound-directed oocyte retrieval (TVOR) was carried out 36 h following kisspeptin-54 injection, and ICSI was performed using fresh sperm from the male partner. One or two embryos were transferred to the uterine cavity 3–5 d following oocyte retrieval. If pregnancy was confirmed, progesterone and estradiol supplementation were provided for luteal phase support until 12 weeks' gestation. Progesterone 100 mg daily im injections (Gestone, Nordic Pharma) were used from the morning following oocyte retrieval until 6 weeks' gestation, then 400 mg twice daily progesterone suppository/pessary (Cyclogest; Actavis) was continued until 12 weeks' gestation. Estradiol valerate 2 mg orally three times daily (Progynova; Bayer) was commenced from the evening of TVOR until 12 weeks' gestation. All women recruited to the study were regarded as being at high risk of OHSS and were routinely screened for the development of early OHSS (assessed on day of embryo transfer 3–5 d after TVOR) and late OHSS (assessed 11 d after embryo transfer). Biochemical pregnancy (serum βHCG > 10 miU/mL) was assessed 11 d following embryo transfer and clinical pregnancy was assessed by ultrasonography at 6 weeks' gestation.

All of the women underwent controlled ovarian stimulation using a recombinant FSH/GnRH antagonist protocol and kisspeptin-54 was used to trigger oocyte maturation as previously described and summarized in Figure 2. On day 2 or 3 of the menstrual cycle, daily sc recombinant FSH injections (Gonal F 112.5 IU, Merck Serono; administered at 0900 h daily), were commenced; pelvic ultrasound scan was performed 5 days after commencing injections to determine ovarian follicular development. Daily sc GnRH antagonist injections (cetrotide, 0.25 mg; Merck Serono; injected at 2100 h daily) were used to inhibit a premature LH surge and were commenced on day 5 of recombinant FSH injections. If serum LH was undetectable (< 0.5 IU/L) on day 7 of recombinant FSH injections, then the dose of cetrotide was halved to 0.125 mg daily. Further ultrasound scans were performed according to follicular size and response to superovulation. When at least three ovarian follicles of at least 18 mm diameter were visible on ultrasound, a sc bolus injection of kisspeptin-54 was administered by a study investigator to trigger oocyte maturation. All doses of kisspeptin-54 were weight adjusted and injection volumes ranged from a minimum of 300 μL to a maximum of 580 μL. Kisspeptin-54 was administered 36 hours prior to oocyte retrieval (between 2030 and 2300 h). Injections of FSH were stopped 12 hours prior to kisspeptin-54. The final dose of GnRH antagonist was administered 24 hours prior to kisspeptin-54. Serum LH, FSH, estradiol, and progesterone were measured immediately before, 12 and 36 hours after kisspeptin-54 injection in all women. Transvaginal ultrasound–directed oocyte retrieval was carried out 36 hours following kisspeptin administration under iv anesthesia (propofol). ICSI was performed in all study cycles using sperm from the male partner to allow for assessment of oocyte maturation. All embryos were graded at day 3 by an independent embryologist, blinded to doses of kisspeptin administered, using the British Fertility Society and Association of Clinical Embryologist embryo grading scheme for cleavage stage embryos, which describes embryos based on cell number, blastomere size, and fragmentation (29). If at day 3 at least two embryos had six or more cells, less than 20% difference in blastomere diameter, and less than 20% fragmentation, they were incubated until day 5 post oocyte retrieval so that the strongest embryos could be identified for transfer. At day 5, embryos were graded for blastocyst expansion (1–6), inner cell mass (A–E) and trophectoderm (A–C) (29). At blastocyst stage, embryos that scored at least 3 for blastocyst expansion and A or B for inner cell mass and trophectoderm were classified as high-quality embryos. One or two embryos of highest quality during morphological assessment were transferred to the uterine cavity 3–5 days following oocyte retrieval.

### Assessment of ovarian hyperstimulation syndrome

All women recruited to the study were regarded as being at high risk of ovarian hyperstimulation syndrome (OHSS). Therefore, women were routinely screened for the development of early OHSS (assessed at embryo transfer 3–5 d following oocyte retrieval) and late OHSS (assessed 11 d following embryo transfer). Women were screened by symptoms (ie, abdominal pain, abdominal bloating, diarrhea, nausea, vomiting, and subjective reduction in urine output), blood analysis (ie, hemaglobin, hematocrit, white cell count, liver function, renal function, and coagulation profile), and ultrasound parameters (ie, ovarian size, presence of free fluid in pouch of Douglas, adnexae, and abdomen or pleural cavity). OHSS was graded according to the criteria of Golan et al (16) with updated categorization by Navot et al (17). OHSS was independently graded by two experienced IVF clinicians external to the study team (S.L. and R.S.), who were provided with blinded data concerning patient symptoms, blood analysis, and ultrasound parameters. In the event of any discrepancy in categorization of OHSS, the more severe classification was used. Women who did not have fresh embryo transfer due to lack of oocyte maturation/fertilization were screened by symptoms alone due to low risk of OHSS.

To safeguard participant well being throughout the study, a clinical decision was requested from the senior IVF study clinician (G.T.) prior to kisspeptin-54 trigger administration in all patients with more than 18 follicles of at least 11 mm, or serum estradiol level greater than 18 000 pmol/L as to whether to proceed with fresh embryo transfer or to cryopreserve all embryos and conduct embryo transfer in a subsequent cycle (segmentation). Women who had segmentation were screened for early OHSS at 5 days following oocyte retrieval but were screened for late OHSS by symptoms alone unless the results of early screening revealed any abnormality on ultrasound or blood parameters.

### Statistical analysis

The study data were summarized using standard descriptive methods. Data analysis was performed by M.S. and D.A.A., who had no involvement in patient management or data collection. Histograms and box plots were used to assess the distributional assumptions, and check for possible outliers. Continuous variables following an approximately normal distribution were summarized using mean and standard deviation. Skewed continuous variables were summarized using median and interquartile range. Categorical variables (binary, ordinal, and multinomial) were presented in terms of frequencies and percentages. All analyses were carried out using Stata statistical software, version 13.1 (StataCorp).

Additional detail regarding study approvals, peptide preparation, and hormonal methodology are presented in Supplemental Methods.

## Results

### Kisspeptin dose allocation and baseline characteristics

The first 15 participants were randomly assigned equally to receive 3.2, 6.4, or 12.8 nmol/kg kisspeptin-54 (n = 5 per group). Kisspeptin-54, 3.2 nmol/kg resulted in a lower rate of oocyte maturation when compared with kisspeptin-54, 6.4 or 12.8 nmol/kg (Supplemental Table 1). Thus, as per the study protocol, remaining participants were randomly assigned to receive 6.4, 9.6, or 12.8 nmol/kg of kisspeptin-54 (n = 15 per group). This adaptive design for dose allocation resulted in the following final subject number for each dose of kisspeptin-54: n = 5, 3.2 nmol/kg; n = 20, 6.4 nmol/kg; n = 15, 9.6 nmol/kg; n = 20, 12.8 nmol/kg (Figure 1). No imbalances in baseline characteristics were observed between the dosing groups other than serum AMH, although an adjusted regression model (data not presented) suggested that this had little influence on the results (see Table 1 for baseline characteristics).

**Table 1.** Baseline Characteristics of Patients Who Received Kisspeptin-54 Trigger

Characteristics	Kisspeptin-54 Dose (nmol/kg)				
	3.2	6.4	9.6	12.8	All Doses
N	5	20	15	20	60
Age, y	30 (28, 31)	30 (28, 31)	32 (30, 33)	31 (28, 34)	31 (28, 33)
Weight, kg	63 (49, 64)	60 (54, 66)	67 (58, 73)	62 (57, 69)	63 (56, 68)
BMI, kg/m^2^	22 (21, 25)	22 (20, 27)	25 (23, 27)	24 (21, 28)	24 (21, 27)
AFC	35 (32, 36)	33 (29, 52)	38 (27, 50)	40 (32, 48)	37 (29, 50)
Serum AMH, pmol/L	35 (29, 50)	48 (37, 67)	66 (41, 68)	53 (43, 68)	50 (39, 68)
Menstrual cycle length, d	28 (28, 40)	32 (30, 35)	35 (30, 41)	33 (30, 70)	33 (30, 40)
Cause of infertility					
PCOS^a^	2 (40%)	4 (20%)	7 (47%)	10 (50%)	22 (37%)
Tubal defect^b^	0 (0%)	4 (20%)	0 (0%)	0 (0%)	4 (7%)
Male factor^c^	1 (20%)	3 (15%)	3 (20%)	4 (20%)	11 (18%)
Other	0 (0%)	1 (5%)	0 (0%)	0 (0%)	1 (2%)
Mixed	0 (0%)	1 (5%)	1 (7%)	3 (15%)	5 (8%)
Idiopathic	2 (40%)	7 (35%)	4 (27%)	3 (15%)	16 (27%)
No. of follicles^d^	32 (28, 33)	30 (26, 35)	34 (22, 39)	39 (29, 45)	34 (26, 40)
No. of follicles ≥ 11 mm^d^	21 (20, 23)	19 (15, 26)	18 (14, 24)	22 (19, 29)	21 (16, 25)
No. of follicles ≥ 14 mm^d^	14 (13, 14)	12 (9, 16)	10 (8, 13)	14 (9, 17)	13 (9, 16)

Abbreviation: PCOS, polycystic ovarian syndrome.

Data are expresses as Median (Lower quartile, Upper quartile) for continuous variables and Total (Percentage) for categorical variables.

a Anovulation due to PCOS.

b Blocked or removed Fallopian tubes.

c Infertility due to a problem with male partner's fertility.

d On final ultrasound scan during controlled ovarian stimulation prior to kisspeptin-54 trigger administration.

### Primary outcome

#### Oocyte maturation

Oocyte maturation (≥ 1 mature oocyte) was observed in 95% (57/60) of women who received kisspeptin-54. Highest oocyte yield (121%) was observed following 12.8 nmol/kg kisspeptin-54, which was +69% (95% confidence interval, –16–153%) greater than following 3.2 nmol/kg (See Supplemental Table 2 for absolute difference in markers of oocyte maturation by kisspeptin-54 dose and Table 2 for oocyte maturation data).

**Table 2.** IVF Outcome Measures Following Kisspeptin-54 Trigger

Outcome Measures	Kisspeptin-54 Dose (nmol/kg)				
	3.2	6.4	9.6	12.8	All Doses
n	5	20	15	20	60
No. of Oocytes	8.8 (5.5)	14.6 (11.1)	11.9 (7.6)	17.5 (10.9)	14.4 (10.0)
No. of mature (M2) oocytes	6.8 (5.6)	11.6 (8.8)	8.3 (6.3)	14.1 (9.9)	11.2 (8.6)
Oocyte maturation rate, %^a^	62 (37)	79 (21)	66 (29)	78 (24)	74 (26)
Oocyte yield, %^b^	53 (41)	86 (49)	86 (74)	121 (119)	95 (85)
No. of 2PN zygotes	5.8 (4.8)	9.4 (7.0)	6.5 (5.6)	11.1 (8.4)	8.9 (7.2)
Fertilization rate, %^c^	68 (39)	76 (29)	74 (33)	73 (21)	74 (28)
No. of patients with embryo transfer	4 (80%)	17 (85%)	13 (87%)	17 (85%)	51 (85%)
No. of cleaved embryos at 3 d post ICSI	5.8 (4.8)	9.4 (7.0)	6.5 (5.6)	11.1 (8.4)	8.9 (7.2)
No. of embryos at day 3 graded as 633 or above	4.4 (3.0)	6.6 (6.4)	4.0 (3.0)	6.9 (5.9)	5.8 (5.4)
No. of patients with day 5 transfer	4 (80%)	15 (75%)	11 (73%)	17 (85%)	47 (78%)
No. of embryos at day 5	5.6 (4.6)	8.6 (7.3)	6.1 (5.9)	10.6 (7.9)	8.4 (7.1)
No. of high-quality embryos (>3A/B) at day 5	1.6 (2.1)	2.4 (2.9)	1.1 (1.4)	1.9 (2.5)	1.8 (2.4)
No. of high-quality embryos (>3A/B) transferred	0.8 (0.8)	0.9 (0.8)	0.7 (0.7)	0.8 (0.7)	0.8 (0.8)
Biochemical pregnancy rate per transfer, %	50.0	64.7	84.6	47.1	62.7
Clinical pregnancy rate per transfer, %	25.0	58.8	76.9	35.3	52.9
Implantation rate, %^d^	25.0 (50.0)	47.1 (45.0)	57.7 (40.0)	29.4 (43.5)	42.2 (44.0)
Live birth rate per transfer, %	25.0	52.9	61.5	29.4	45.1

Abbreviations. general comments, footnotes.

Data are presented as Means (SD) for continuous variables and Total (Percentages of N) for categorical variables.

a Oocyte maturation rate is the percentage of oocytes collected which were mature.

b Oocyte yield is the percentage of mature oocytes collected from the number of follicles ≥ 14 mm in diameter on the final ultrasound scan prior to kisspeptin-54 trigger administration.

c Fertilization rate is the percentage of mature oocytes that fertilize following intracytoplasmic injection with sperm (ICSI).

d Implantation rate is defined as the percentage of embryos transferred that implant on assessment by ultrasound at 6 wk of gestation.

### Secondary outcomes

#### Embryo transfer

Oocyte maturation did not occur in three women and three additional women had mature oocytes retrieved but fertilization did not take place following ICSI. Thus, embryo formation occurred in 90% (54/60) of women who received kisspeptin-54. A clinical decision for segmentation was made in three women. Thus, 51 of 60 women had fresh embryo transfer in this study (see Table 3 for summary of stage of IVF outcome achieved at each dose of kisspeptin-54).

**Table 3.** Summary of Patient Response Following Kisspeptin-54 Triggering

Kisspeptin-54 Dose	3.2	6.4	9.6	12.8	Total
N	5	20	15	20	60
≥ 1 mature oocyte retrieved	4	20	14	19	57
≥ 1 2PN zygote formed^a^	4	18	13	19	54
Segmentation^b^	0	1	0	2	3
No. of patients with embryo transfer	4	17	13	17	51
No. of patients with day 5 transfer^c^	4	15	11	17	47
High-quality embryo transfer^d^	3	13	9	13	37
Biochemical pregnancy at 11 d	2	11	11	8	32
Clinical pregnancy at 6 wk	1	10	10	6	27
Miscarriage/still birth	0	1	2	1	4
Live birth	1	9	8	5	23

Data are presented as the number of patients completing each criterion.

a 2PN is two pronuclear zygote assessed on day following oocyte retrieval.

b Segmentation refers to cryopreservation of all embryos and embryo transfer in a subsequent frozen cycle due to high risk of OHSS.

c Embryo transfer on day 5.

d Transfer of at least one high-quality embryo. High-quality embryos were blastocyst embryos graded on day 5 as being ≥ 3A or B in quality (30).

#### Pregnancy rates

At all doses of kisspeptin-54 tested, biochemical pregnancy, clinical pregnancy, and live birth rates per transfer (n = 51) were 63, 53, and 45%, respectively. Highest pregnancy rates were observed following 9.6 nmol/kg kisspeptin-54 (85, 77, and 62%, respectively) with an implantation rate of 57.7%. (See Table 2 for pregnancy and implantation rates following kisspeptin-54).

#### Rates of OHSS

Of the 60 women in the study, only three (5%) were diagnosed with mild early OHSS and one with mild late OHSS (2%), but no woman had moderate, severe, or critical OHSS and no woman required medical intervention for OHSS. None of the three women who had segmentation because of a very high risk of OHSS prior to kisspeptin-54 administration had any features of early OHSS on screening (see Table 4 for full data on OHSS screening).

**Table 4.** OHSS in High Risk-Women Following Kisspeptin-54 Trigger

	Early OHSS		Late OHSS	
OHSS Symptomatology				
No. of patients screened by OHSS symptoms	n = 60		n = 60	
≥1 symptom potentially consistent with OHSS	9 (15%)		11 (18%)	
No. of patients requiring medical intervention or hospitalization for OHSS	0		0	
Sonographical screening				
No. of patients screened with pelvic ultrasound	n = 54		n = 51	
	Left	Right	Left	Right
Mean ovarian volume (mls)	45 (35)	50 (32)	16 (11)	19 (15)
Max ovarian diameter (mm)	47 (13)	48 (12)	36 (8)	38 (10)
No. of patients with maximum ovarian diameter >5 cm	35 (58%)		13 (22%)	
No. of patients with maximum ovarian diameter >8 cm	7 (12%)		9 (15%)	
No. of patients with pleural effusion	0		0	
No. of patients with free fluid in abdomen	0		0	
No. of patients with fluid in POD/adnexa	26 (43%)		8 (13%)	
Blood parameters				
No. of patients screened with blood analysis	n = 53		n = 50	
No. of patients with hematocrit >45%	0		0	
No. of patients with white cell count >15 × 10^9^/L	0		1	
No. of patients with ALT or AST >2 × ULN	1 (2%)		1 (2%)	
No. of patients with total protein >80 g/L	2 (3%)		4 (7%)	
No. of patient with creatinine >110 μmol/L	0		0	
No. of patients with OHSS^a^				
Normal	57 (95%)		59 (98%)	
Mild	3 (5%)		1 (2%)	
Moderate	0		0	
Severe	0		0	
Critical	0		0	

Abbreviations: POD, Pouch of douglas; ALT, Alanine transaminase; ULN, Upper Limit of Normal.

a Diagnosis of OHSS was performed by two experienced IVF physicians independent of the study team provided with blinded data according to the criteria (16) with updated categorization of severe and critical OHSS (17).

#### Reproductive hormone levels

Reproductive hormone levels were elevated at 12 hours following kisspeptin-54 administration and returned toward baseline levels at 36 hours following kisspeptin-54 administration (see Supplemental Figure 1 for reproductive hormone levels following kisspeptin-54 trigger). Median LH levels at 12 hours following kisspeptin-54 trigger administration were highest following 12.8 nmol/kg and lowest following 3.2 nmol/kg kisspeptin-54.

### Adverse events

Kisspeptin-54 administration was well tolerated in all 60 women in this study. Six adverse events occurred that are established complications of IVF treatment/pregnancy (two patients experienced ectopic pregnancy; three women experienced miscarriage at 12, 12, and 14 weeks' gestation; and one pregnancy resulted in still birth at 25 weeks' gestation).

## Discussion

In this study we have shown that the use of kisspeptin-54 to trigger oocyte maturation in a cohort of women at high risk of OHSS resulted in high rates of oocyte maturation, high implantation rates, and no cases of clinically significant OHSS.

We selected women for inclusion into the study with either a serum AMH level at least 40 pmol/L or AFC greater than 23 on ultrasound given that both of these markers have been shown to accurately identify women at high risk of developing OHSS during IVF treatment. The risk of moderate to severe OHSS is increased more than 5-fold, from 6% in women with a serum AMH less than 40 pmol/L to 33% in women with an AMH of at least 40 pmol/L (14). Similarly, in patients with an AFC greater than 23, the risk of moderate to severe OHSS is increased 4-fold to 8.6% (15). Indeed, the risk of OHSS increases linearly with AFC, increasing to more than 20% in women with an AFC greater than 40 (15). In our cohort, 75% of patients had an AMH of at least 40 pmol/L, all patients had an AFC greater than 23, and 42% had an AFC greater than 40.

Once women undergo ovarian stimulation during IVF treatment, the number of follicles at least 11 mm in size on the day of trigger administration is predictive of the risk of OHSS. More than 14 such follicles suggests a high risk of OHSS, and more than 25 follicles suggests an extremely high risk of OHSS (18, 19). In our study, 88% of women had more than 14 follicles and 28% of women had more than 25 follicles, confirming our inclusion criteria had successfully identified a patient cohort at high risk of OHSS.

According to the criteria of Jayaprakasan et al (15), we might have expected 27% of the patients in our study (16/60) to have developed moderate to severe OHSS had we used established triggers of oocyte maturation. However, no cases of moderate, severe, or critical OHSS were detected using kisspeptin-54 to trigger oocyte maturation.

Papanikolaou and colleagues (20) identified a threshold level of 18 follicles of at least 11 mm or a serum estradiol level greater than 5000 ng/L (> 18 355 pmol/L) to confer an 83% sensitivity and 84% specificity for identifying women at risk of developing severe OHSS. Thus, to safeguard participant well being throughout this study of patients at high risk of developing OHSS, a clinical decision was requested from the senior IVF study clinician (G.T.) prior to kisspeptin-54 trigger administration in all patients with more than 18 follicles of at least 11 mm or a serum estradiol greater than 18 000 pmol/L on the day of kisspeptin-54 trigger administration as to whether to proceed with fresh embryo transfer or to recommend segmentation (cryopreservation of all embryos with embryo transfer in a subsequent cycle). Although nearly two thirds of women in our patient cohort had more than 18 follicles of at least 11 mm on the day of kisspeptin-54 trigger administration, a clinical decision for segmentation was only made in three women at very high risk of OHSS. The total number of follicles on the final ultrasound scan prior to trigger administration in these three women was 53, 58, and 68 and the number of follicles of at least 11 mm in size was 18, 47, and 39, respectively. Serum estradiol levels on the morning of trigger administration were 46 524, 26 378, and 11 768 pmol/L, respectively. None of the three women who were treated with segmentation for extremely high risk of OHSS subsequently developed any features of early OHSS on screening following kisspeptin-54 triggering. Two of the three women went on to have healthy clinical pregnancies established following their subsequent frozen embryo transfers, whereas the remaining woman had only a biochemical pregnancy confirmed.

Fifteen of the sixty women recruited to this study had previously been treated with an hCG-triggered IVF cycle prior to entry to the study. During their previous IVF cycle using hCG to trigger oocyte maturation, three of these 15 women (20%) had developed severe OHSS requiring admission to hospital for medical intervention or intensive care support. None of these three women had any evidence of OHSS during their kisspeptin-54-triggered cycle and two of these three patients went on to have healthy pregnancies during their study cycle.

Oocyte maturation occurred in 95% (57/60) of women, and embryo formation in 90% (54/60) of women following kisspeptin triggering. In two of the three women without oocyte maturation, an insufficient increase in serum LH was detected at 12 hours following trigger administration of less than 0.7 IU/L, compared with a mean increase of 8.1 IU/L in remaining patients with oocyte maturation. Three additional women had oocyte maturation, but fertilization did not occur following ICSI. One of these three women without embryo formation had nine mature oocytes retrieved, but an inadequate sperm sample was produced on the day of ICSI. In two of the six women without embryos available for transfer a premature increase in serum progesterone (> 10 nmol/L) was detected prior to kisspeptin-54 trigger administration following prolonged phases of controlled ovarian stimulation (16 and 22 d of recombinant FSH). A premature increase in serum progesterone to greater than 1.5ng/mL (4.8 nmol/L) during IVF cycles has previously been reported to be associated with adverse outcomes during IVF treatment.

Kisspeptin-54 was well tolerated in all participants with no adverse events associated with the kisspeptin-54 injection. Two of 31 biochemical pregnancies (6.5%) were ectopically situated, which is comparable to the rate encountered using a GnRH agonist trigger of 7.4% (4/54) in an equivalent patient group (age < 35 y; AFC > 23; BMI < 29 kg/m^2^; ICSI) during 2011–2014 in our unit and other reported studies (21).

The most widely currently used strategy for the avoidance of OHSS in high-risk populations is to trigger oocyte maturation with a GnRH agonist trigger in place of hCG. The use of GnRH agonist triggers is associated with lower rates of OHSS than following hCG, however, recent case reports suggest that use of GnRH agonist triggers, even in the absence of supplemental hCG for luteal phase support and with segmentation, does not eliminate the occurrence of severe OHSS in high-risk populations (22–24). The use of GnRH agonists to trigger oocyte maturation during IVF treatment also exacerbates luteal phase insufficiency, resulting in reduced implantation rates (11). A number of strategies have been used in an attempt to improve implantation rates following GnRH agonist triggering, including the addition of high-dose sex-steroid replacement during the luteal phase (25, 26), the use of a small dose of hCG either at the time of trigger (27), or during the early luteal phase (6, 19). However, the addition of even a small dose of hCG to high-risk populations can result in severe OHSS in up to a quarter of women (6). The pregnancy rates observed in our study compare favorably with currently used triggers of oocyte maturation. According to the most recently published Human Fertilisation Embryology Authority data of all registered treatment centers in the United Kingdom, the clinical pregnancy rate in women age 34 years or younger using currently available triggers of oocyte maturation was 41.8% and live birth rate per transfer 37.5% (1) At all doses of kisspeptin-54 examined, we observed a 52.9% clinical pregnancy rate and a 45.1% live birth rate per transfer. Furthermore, highest pregnancy rates were observed following 9.6 nmol/kg kisspeptin-54, which resulted in a 76.9% clinical pregnancy rate and 65.1% live birth rate per transfer. However, it is not known whether these excellent pregnancy rates could be obtained with alternate luteal phase regimens.

An ideal trigger for inducing oocyte maturation in patients undergoing IVF treatment would yield high rates of oocyte maturation, allow for high implantation rates, yet carry a low risk of inducing OHSS (28). In this study we observed that using kisspeptin-54 to trigger oocyte maturation in women at high risk of OHSS resulted in high rates of oocyte maturation, high implantation rates, and no clinically significant OHSS. These results are promising and additional large randomized studies are now warranted to directly compare the efficacy and safety of kisspeptin-54 vs currently used triggers to verify the optimal trigger of oocyte maturation in patients at high risk of developing OHSS during IVF treatment.
